# How Do We Combat Bogus Medicines in the Age of the COVID-19 Pandemic?

**DOI:** 10.4269/ajtmh.20-0903

**Published:** 2020-08-18

**Authors:** Wubshet Tesfaye, Solomon Abrha, Mahipal Sinnollareddy, Bruce Arnold, Andrew Brown, Cynthia Matthew, Victor M. Oguoma, Gregory M. Peterson, Jackson Thomas

**Affiliations:** 1 Health Research Institute, University of Canberra, Canberra, Australia;; 2 University of Canberra, Canberra, Australia;; 3 Mekelle University, Mek’ele, Ethiopia;; 4 Therapeutics Goods Administration, Canberra, Australia;; 5 Canberra Law School, University of Canberra, Canberra, Australia;; 6 IntraHealth International, Durham, North Carolina;; 7 University of Tasmania, Hobart, Australia

## Abstract

The COVID-19 pandemic has brought concurrent challenges. The increased incidence of fake and falsified product distribution is one of these problems with tremendous impact, especially in low- and middle-income countries. Up to a tenth of medicines including antibiotics and antimalarial drugs in the African market are considered falsified. Pandemics make this worse by creating an ecosystem of confusion, distraction, and vulnerability stemming from the pandemic as health systems become more stressed and the workload of individuals increased. These environments create opportunities for substandard and falsified medicines to be more easily introduced into the marketplace by unscrupulous operators. In this work we discuss some of the challenges with fake or falsified product distribution in the context of COVID-19 and proposed strategies to best manage this problem.

## INTRODUCTION

COVID-19 has revealed weaknesses and vulnerabilities in global and regional health governance, with the proliferation of “fake health news” and the Surgisphere scandal exacerbating the dissemination of fake medicines.^1–4^ These weaknesses are of particular concern in developing countries with inadequate health infrastructures.^5,6^

Research into finding an effective treatment for COVID-19 has continued unabated. Although some observational studies showed early promise with chloroquine and hydroxychloroquine, subsequent randomized trials revealed the lack of clinical benefit on these drugs.^7,8^ The discontinuation of the major trials on hydroxychloroquine—recovery^9^ and solidarity^10^—further strengthens the notion that it may not be effective, especially in severe COVID-19 cases. Subsequently, randomized trials revealed positive outcomes associated with remdesivir and dexamethasone use in people with severe COVID-19.^11,12^ Remdesivir was associated with shorter time to recovery,^11^ whereas dexamethasone significantly cut mortality rates in people on oxygen support.^12^ COVID-19 management protocols in different jurisdictions are modified accordingly, although firm evidence is needed to clearly understand the benefit of these drugs and to whom. On top of that, accelerated vaccine developments are underway in various countries, with 21 of them currently under clinical evaluation and one in phase III testing.

Given the urgency associated with finding the magic bullet for COVID-19, research has become increasingly challenging and is resulting in self-examination by the scientific community and the wider public about the governance of fast-tracked research.^13^ The hype with chloroquine and hydroxychloroquine at the beginning was associated with misinformation^14^—sometimes in the form of “fake health news”—and incidents of quarantine-based supply chain disruption and stockpiling.^15^

For the Global South, the pandemic exacerbates the existing proliferation of substandard and falsified medical products, and these trends are likely to continue as effective drugs continue to emerge from ongoing trials. The WHO defines substandard products as “authorized medical products that fail to meet quality standards or specifications, or both” and falsified products as “products that deliberately/fraudulently misrepresent identity, composition, or source.”^16^ Considering less than 30% of regulatory agencies in the world can ensure the adequacy of medicines and vaccines,^17^ pandemics of COVID-19 proportion would inevitably present enormous regulatory challenges. Health emergencies can create an environment of confusion, distraction, and vulnerability stemming from the pandemic, as health systems become more stressed and the workload of individuals increased. These environments create opportunities for substandard and falsified medicines to be more easily introduced into the marketplace by unscrupulous operators. It is worth noting that supply chains are fragile even in normal times,^18^ and the COVID-19 pandemic made the vulnerabilities with the supply chains even more noticeable. The supply chain disruption caused by the reduced production and export of some medicines owing to closure of China’s active pharmaceutical ingredient and raw material supply revealed this vulnerability.^19^ This has stimulated debate on the need to diversify the sources of pharmaceutical supply to ensure continuity of supply across the globe.^20^ There are ongoing industry efforts to support initiatives to maximize domestic productions of raw materials to prevent disruption to the supply chain in the future.^19–21^ These problems particularly need further attention in fragile healthcare systems and are highly dependent on the import of medicines to cover their domestic demand.

Falsified medical products may contain no active ingredient, the wrong active ingredient, or the wrong amount of the correct active ingredient.^16^ The prevalence of falsified medicines spans from 1% in developed settings to 10% in the Global South.^22,23^ Based on more than $4.3 billion worth of substandard and falsified products seized by border security agents from 2014 to 2016, over 35% of the products were antibiotics and 9% were antimalarials.^24^ Counterfeiters also target treatments for diabetes, epilepsy, heart diseases, allergy, blood pressure, cancer, and stomach ulcers.^25^

Falsified and substandard medicines are deliberately produced mostly for monetary gain and do not produce the required therapeutic benefits.^16,26–28^ Evidence shows that the use of falsified medicines is associated with serious health outcomes, including mortality both in developing and developed countries.^27^ In low- and middle-income countries (LMICs) alone, falsified medicines cost economies up to US$200 billion for additional care following treatment failure.^26,29^ Nearly half of the WHO reports on falsified medical products (42%) originate from the African region, and many are linked with antimalarial drugs. In sub-Saharan Africa, substandard and falsified antimalarials are estimated to cost US$38.5 million (US$21.4–US$52.4 million) and 116,000 (64,000–158,000) additional deaths^16,29^—which can be prevented.

### Falsified products in the context of COVID-19.

Although a true estimate on the impact of falsified medical products during COVID-19 is yet to be made, the pandemic has fueled a global surge in the production and distribution of falsified medicines and medical supplies^25^—resulting in a multifold global crisis. This is evident from recent operations by The International Criminal Police Organization that captured millions of units of counterfeit pharmaceuticals (antiviral medications, antimalarial chloroquine, vitamin C, painkillers, and antibiotics), coupled with several reports on the counterfeit medical supplies (mostly surgical masks and coronavirus testing kits) from around the globe. Similarly, in Africa, the early days of the pandemic have seen a spike in the demand for chloroquine and hydroxychloroquine that subsequently led to a surge in their falsified version.^30^ The WHO global surveillance and monitoring system on substandard and falsified medical products received nine reports of confirmed falsified chloroquine products from three countries—Cameroon, Democratic Republic of Congo, and Niger between March 31, 2020 and April 2, 2020.^31^ This is a worrisome development for a continent with a long-standing problem of falsified antimalarial drugs.^32^ Looking at the trends of a surge in falsified products and in the absence of proper regulation, drugs such as dexamethasone may have a similar fate. Therefore, regulatory agencies should have additional oversight on the distribution of products containing this drug to ensure their quality, particularly in settings where product regulation is weak. The trend is also likely to be much higher this year as the demand for important drugs exceeds the supply, following COVID-19–led lockdowns in India and China (two largest producers of medical products).^8,30^

African nations are largely spending their scarce resources on COVID-19 containment measures, aiming to prevent large-scale disease dissemination that can lead to huge health, social, and economic crises. This will undoubtedly leave them with limited capacity to tackle other parallel emergencies. Falsified medicines tend to emerge when access to medicines is constrained, when the pharmaceutical governance is weak, where there is limited technical capacity to monitor products throughout the supply chain, and when there is lack of adequate financial and political commitments.^33^ These standards come under the purview of the national regulatory system. A looming recession on the horizon is also likely to stimulate social tolerance for substandard and falsified medical products. Strong regulatory frameworks are, therefore, essential in enabling regulatory authorities to effectively combat the distribution of falsified medicines. Corrupt and under-resourced systems, coupled with limited capacity for oversight, allow falsified medicine to easily reach the end user.^32^ High-income countries have a strict regulatory framework, technological means, and financial resources to detect and limit the distribution of falsified and counterfeited products. The penetration of counterfeit products is generally higher in LMIC settings. There is a risk that when the COVID-19 pandemic further spreads in LMICs, there will be an increased potential for the distribution of falsified and counterfeit medicines, something the international community needs to be alert to and work against.

### Ongoing efforts to tackle distribution of falsified products.

Given the complexity of the pharmaceutical distribution and supply chain, vulnerabilities could occur at any stage across the supply chain, endangering patients around the world.^34^ Building collaborations across countries and private and public agencies is required to strengthen the regulatory footprint at national and international levels. The U.S. Food and Drug Administration and the European Medicines Agency are trialing to implement various technologies to track the steps involved in the supply chain. In Africa, a health information technology/big data startup RxAll has come up with a handheld scanner, which collects the spectral signature of a drug and then compares it with a cloud-based database and sends information to an application on the phone. Although field detection technologies could play a role in intercepting substandard and falsified products, their cost feasibility and when to effectively use them across the supply chain continuum remain to be understood.^35,36^ Also, this seems to be a palliative approach to a multifactorial systemic problem.

There are some global efforts to improve access and quality of medicines. The United States Agency for International Development (USAID) Global Health Supply Chain Program is exploring innovations based on prior experiences to devise more efficient supply chains aiming to implement global supply chain standards (GS1) for improved cost, efficiency, and availability of health commodities worldwide.^37^ Furthermore, some programs specifically aim to strengthen regulatory agencies in LMICs with the ultimate goal to ensure sustainable access to appropriate, safe, affordable, and quality-assured medicines.^38,39^ This also includes supply chain strengthening activities undertaken by USAID, UNICEF, the United Nations Population Fund, the Global Fund, Bill & Melinda Gates Foundation, and others that focus on supporting the end to end product traceability supported by the use of novel technologies. However, recent attempts to halt funding arrangements for the WHO could be a serious threat to global health security, especially in the middle of a catastrophic global pandemic.

## CONCLUSION

In summary, the availability and distribution of falsified products is an age-old problem, with higher penetration in LMICs, a situation that is likely to get worse as disruptions place greater stresses on regulatory and supply chain processes. In the age of COVID-19, there is an ongoing need to ensure adequate supplies of medicines and medical equipment, not only for meeting the needs of the pandemic but also for other health needs. In meeting these needs, governments, pharmaceutical regulatory agencies, and associate supply chains must have practical and financially supported strategies to ensure quality-assured medicines are made available for the determined need. More importantly, it is imperative medicines regulatory authorities and relevant stakeholders implement robust authentication and procurement processes to ensure quality medicines supply.^40^ In line with this, we propose the following set of strategies can be cascaded to relevant stakeholders (Table 1) to ensure the availability of quality medicines during the time of COVID-19 and beyond.

**Table 1:** Cascaded approach to prevent falsified products

Stakeholder	Roles
International regulatory agencies	Strengthening national and international pharmaceutical governance (agencies such as INTERPOL have intercepted significant falsified products, which should continue)
	Adequate funding for monitoring distribution of falsified products
	Initiation and/or assistance in coordinating the information-sharing process among regulators in different regions and countries
Supply agencies	Ensuring security of supply chains through implementation of good distribution and warehousing practice.
	Adequate and continuous supply of standard drugs can avert the bad ones from filling the vacuum in times where there is a heightened fear and desperation for treatments
	Good procurement practice with an extra focus on quality
Local regulatory agencies	Regulatory agencies are expected to fulfill the Global Benchmarking Tool, which has more than 200 indicators to measure effectiveness of regulatory functions in a country. At this stage, Tanzania is the only country that achieved such a milestone in Africa,^17^ emphasizing on the need for other African nations to leverage this local experience for implementation in their respective settings
	Have the responsibility to make sure there is a routine inspection on their borders in relation to the entry of new medicines and medical products
	Implementation of legislative changes restricting counterfeit drugs
	Improved investment capital and infrastructure to encourage small- and medium-sized drug manufacturing companies to meeting international standards
	Strong political will to enforce regulations
Health ministries	Creating awareness among citizens regarding medicine quality through their public health section
Technological companies	Implementation of detection technologies to enable easy identification of falsified products, when affordable and appropriate
	Social media platforms should be held accountable for the dissemination of fake health news, with a more proactive identification and removal of fake health news provided by armchair epidemiologists, vendors of bogus cures, and citizen journalists. This requires working closely with consumer protection agencies across the globe and with qualified public health officials to take quick and decisive steps to remove inappropriate and inaccurate content, and to prevent it appearing in the first place
Researchers and health professionals	The hype associated with finding a cure for COVID-19 has changed the way research is communicated to the public, with potentially flawed or premature research findings being hastily released. Researchers and health professionals need to play a greater role in controlling the narrative surrounding COVID-19 research
	Continued engagement with the communities through education, training, and public discourse can reduce harm and save lives
	Health professionals should always be vigilant of newer medicines joining the market, especially those with increased relevance due to the pandemic
Citizens	The pandemic response cannot be effective without active participation of the people. It is important to heed advices by health experts, especially in relation to drugs, and to differentiate between fact-based reporting and nonfactual claims particularly when it comes to medicine-related information.^41^
